# Supplementary material for: Retinoic Acid Receptor Is a Novel Therapeutic Target for Postoperative Cognitive Dysfunction
Source: Pharmaceutics. 2023 Sep 13;15(9):2311. doi: 10.3390/pharmaceutics15092311 (PMC10538227; doi:10.3390/pharmaceutics15092311)
Supplement: Supplementary file 1 [file pharmaceutics-15-02311-s001.zip › pharmaceutics-2559447-supplementary.pdf]

Table S1. Details of POCD landmark genes for the establishment of CMap model of POCD.

Genes symbol	Alteration in POCD	Summary of Gene Function or Product
VNN1	Up	One of Vanin family protein involved in hematopoietic cell trafficking and enables pantetheine hydrolase activity
LY6G5C	Up	A lymphocyte antigen 6 family member involved in identical protein binding
NEK3	Up	NIMA related kinase 3 involved in phosphorylation of VAV2 guanine nucleotide exchange factor, paxillin, and activation of the RAC1 GTPase
ZFYVE16	Up	Zinc finger FYVE-type protein involved in membrane trafficking in the endosome
ITFG1	Up	Located in extracellular exosome
HDAC5	Up	Histone deacetylase involved in alteration of chromosome structure and transcription factor access to DNA
CRYZ	Up	Taxon-specific crystallin protein with NADPH-dependent quinone reductase activity
NPY1R	Up	A G-protein-coupled receptor superfamily involved in function of neuropeptide Y and peptide YY
VPS41	Up	Human ortholog of yeast Vps41 protein involved in the formation and fusion of transport vesicles from the Golgi
CUL5	Up	Cullin involved in ubiquitin protein ligase binding activity
EHMT1	Up	A histone methyltransferase involved in cell cycle transition
ARL15	Up	Predicted to enable GTP binding activity and GTPase activity and located in extracellular exosome
MAP4K5	Up	A member of the serine/threonine protein kinase involved in activate Jun kinase and function in stress response
TBXAS1	Up	Thromboxane A synthase involved in pathophysiological processes like hemostasis, cardiovascular disease, and stroke
DAZL	Down	Potential RNA binding proteins that are expressed in prenatal and postnatal germ cells of males and females
ZFR	Down	RNA-binding protein involved in nucleocytoplasmic shuttling of another RNA-binding protein, Staufen homolog 2, in neurons
RTF1	Down	Gene locus involved in regulation of transcription elongation and chromatin remodeling
NAA15	Down	Auxillary subunit of the N-terminal acetyltransferase A complex involved in the acetyl group transfer on nascent polypeptides
SMARCE1	Down	Part of SWI/SNF complex involved in transcriptional activation of genes normally repressed by chromatin
MXI1	Down	Oncogenic transcription factor involved in negatively regulate MYC function
OXSR1	Down	Ser/Thr protein kinase family of proteins involved in response to environmental stress and regulating actin cytoskeleton
RPL39L	Down	Encode a protein sharing high sequence similarity with ribosomal protein L39
CAND1	Down	Regulator of Cullin-RING ubiquitin ligases involved in ubiquitinylation of proteins degraded by the Ub proteasome system
OR1E1	Down	Olfactory receptors involved in neuronal response to perception of smells
OLFML2B	Down	Olfactomedin domain-containing protein
PPP5C	Down	Serine/threonine phosphatase involved in response to hormones or cellular stress and breast cancer development
PARL	Down	Intramembrane serine proteases involved in mitochondrial remodeling and apoptosis
POLR2K	Down	Subunits of RNA polymerase II involved in synthesizing messenger RNA in eukaryotes

PRPF3	Down	Pre-mRNA processing factor 3 involved in retinitis pigmentosa-18
PSG5	Down	Pregnancy-specific glycoproteins mainly produced by the placental syncytiotrophoblasts during pregnancy
WNK1	Down	WNK subfamily of serine/threonine protein kinases involved in the transport of sodium and chloride ions as blood pressure regulator
EIF2AK2	Down	Serine/threonine protein kinase involved in innate immune response against multiple DNA and RNA viruses
BMP2K	Down	human homolog of mouse BMP-2-inducible kinase involved in in skeletal development and patterning
RPL26L1	Down	Alternative splicing results in multiple transcript variants encoding the same protein
ARHGEF6	Down	Cytoplasmic protein that activates the Ras-like family involved in Rho-dependent signals
U2SURP	Down	Enables RNA binding activity
DNAJC13	Down	Dnaj protein family member involved in in clathrin-mediated endocytosis
SMC4	Down	Involved in changes in chromosome structure during mitotic segregation of chromosomes
ATP9B	Down	Predicted to enable ATPase-coupled intramembrane lipid transporter activity
DDX6	Down	A DEAD box protein family member involved in translation suppression, mRNA degradation and microRNA-induced gene silencing
DNASE2B	Down	Share considerable sequence similarity to and is structurally related to DNase II
SREK1	Down	Serine/arginine-rich (SR) splicing protein interacted with other SR proteins to modulate splice site selection
NPIPA1	Down	Predicted to be involved in mRNA transport and protein transport
OAT	Down	Mitochondrial enzyme ornithine aminotransferase involved in converting arginine and ornithine into the major excitatory and inhibitory neurotransmitters glutamate and GABA
CDKL1	Down	A CDC2-related serine/threonine protein kinases member involved in accumulating primarily in the nucleus
USP15	Down	A USP family member involved in transforming growth factor beta signalling through deubiquitination of receptor-activated SMAD transcription factors
SEC24B	Down	A cargo-binding component of the COPII vesicle involved in the transport of secretory proteins from the endoplasmic reticulum to the Golgi apparatus
ATXN2	Down	Encoded cytoplasmic protein is involved in endocytosis, and modulates mTOR signals, modifying ribosomal translation and mitochondrial function
PIP5K1A	Down	Enables 1-phosphatidylinositol-4-phosphate 5-kinase activity and kinase binding activity
CKS1B	Down	Bind to the catalytic subunit of the cyclin dependent kinases and involved in their biological function
SENP6	Down	Remove the tail sequences of the UBL precursors and display isopeptidase activity for deconjugation of SUMO-conjugated substrates
HNRNPC	Down	Act as a tetramer and is involved in the assembly of 40S hnRNP particles
HMGN2	Down	Bind nucleosomal DNA and is associated with transcriptionally active chromatin
UFM1	Down	A ubiquitin-like protein that is conjugated to target proteins by E1-like activating enzyme UBA5 and E2-like conjugating enzyme UFC1
GPR65	Down	Enables G protein-coupled receptor activity and involved in actin cytoskeleton reorganization; activation of GTPase activity; and positive regulation of stress fiber assembly
